# Pleiotropic activation of endothelial function by angiotensin II receptor blockers is crucial to their protective anti-vascular remodeling effects

**DOI:** 10.1038/s41598-022-13772-3

**Published:** 2022-06-13

**Authors:** Arash Y. Tehrani, Zoe White, Lin Wei Tung, Roy Ru Yi Zhao, Nadia Milad, Michael A. Seidman, Elodie Sauge, Marine Theret, Fabio M. V. Rossi, Mitra Esfandiarei, Casey van Breemen, Pascal Bernatchez

**Affiliations:** 1 Centre for Heart Lung Innovation, University of British Columbia, Vancouver, BC Canada; 2 Department of Anesthesiology, Pharmacology and Therapeutics, University of British Columbia, 2176 Health Sciences Mall, Room 217, Vancouver, BC V6T 1Z3 Canada; 3 School of Biomedical Engineering and Department of Medical Genetics, University of British Columbia, Vancouver, BC Canada; 4 Laboratory Medicine Program, University Health Network, Toronto, ON Canada; 5 Department of Biomedical Sciences, College of Graduate Studies, Midwestern University, Glendale, AZ USA

**Keywords:** Aneurysm, Aortic diseases

## Abstract

There are no therapeutics that directly enhance chronic endothelial nitric oxide (NO) release, which is typically associated with vascular homeostasis. In contrast, angiotensin II (AngII) receptor type 1 (AT1R) blockers (ARBs) can attenuate AngII-mediated oxidative stress, which often leads to increased endothelial NO bioavailability. Herein, we investigate the potential presence of direct, AngII/AT1R-independent ARB class effects on endothelial NO release and how this may result in enhanced aortic wall homeostasis and endothelial NO-specific transcriptome changes. Treatment of mice with four different ARBs induced sustained, long-term inhibition of vascular contractility by up to 82% at 16 weeks and 63% at 2 weeks, an effect reversed by L-NAME and absent in endothelial NO synthase (eNOS) KO mice or angiotensin converting enzyme inhibitor captopril-treated animals. In absence of AngII or in tissues with blunted AT1R expression or incubated with an AT2R blocker, telmisartan reduced vascular tone, supporting AngII/AT1R-independent pleiotropism. Finally, telmisartan was able to inhibit aging- and Marfan syndrome (MFS)-associated aortic root widening in NO-sensitive, BP-independent fashions, and correct aberrant TGF-β signaling. RNAseq analyses of aortic tissues identified early eNOS-specific transcriptome reprogramming of the aortic wall in response to telmisartan. This study suggests that ARBs are capable of major class effects on vasodilatory NO release in fashions that may not involve blockade of the AngII/AT1R pathway. Broader prophylactic use of ARBs along with identification of non-AngII/AT1R pathways activated by telmisartan should be investigated.

## Introduction

The vascular endothelium is a complex monolayer of specialized cells that normalize vascular tone, leukocyte adhesion^1^, plasma protein leakage^2,3^, smooth muscle cell proliferation^4^, and of sub-endothelial tissue homeostasis^5^. Endothelial function, a term commonly used to describe the vasodilatory and protective properties of the endothelium, is mostly sustained by nitric oxide (NO), prostacyclin (PGI_2_), and endothelium-derived hyperpolarization (EDH)^6^. The bioavailability of NO, the predominant endothelial vasodilator and marker of endothelial function, is tightly linked to vascular homeostasis whereas loss of protective endothelial function and lower NO bioavailability have been observed in the pathogenesis of various cardiovascular diseases, such as hypertension, atherosclerosis and aortic aneurysm^7^. The constitutively-expressed endothelial NO synthase (eNOS) is the main source of protective vascular NO in response to shear stress or agonist-induced stimulation^8^. Thus, approaches that improve eNOS regulation and endothelial function as a whole are highly relevant to treatment of vascular disease^9–11^.

Statins have pleiotropic, endothelial function-enhancing properties although whether this plays a significant part in their effect on cardiovascular disease outcomes is not known^12^. ACE inhibitors (ACEi) and angiotensin II (AngII) receptor type I (AT1R) blockers (ARBs) have often been linked to improved NO bioavailability^13^ as they can prevent the oxidative stress-enhancing effects of AngII through AT1R blockade and reduce NO scavenging^14^. However, whether ARBs have direct class effects on the vasodilatory properties of the endothelium has never been reported. We have shown that ARB losartan has retained its anti-aortic root widening effects in Marfan syndrome (MFS) mice with blunted AT1R receptor expression^15^, along with high levels of endothelial function activation during chronic losartan^16^ and valsartan^17^ treatment, highlighting the potential presence of AngII/AT1R-independent pleiotropic. In the present report, we show in mice that ARBs share the ability to increase endothelial function for months that far exceed that observed with angiotensin converting enzyme inhibitor (ACEi) captopril on a same BP-lowering level. Furthermore, the pleiotropic ARB telmisartan can act directly reduce vascular tone in isolated aortic vessels free of AngII or normal AT1R/2 signaling ex vivo within minutes, an effect completely abolished by L-NAME or use of eNOS KO animals. We also show that NO release triggered by telmisartan is essential to its pro-aortic stability effect in settings of age- and MFS-associated aortic root widening, and that it promotes an eNOS-specific transcriptome reprogramming of the vasculature. Overall, our provide evidence that ARBs are capable of major class effects on NO-dependent endothelial function independently of their capacity of blocking classic AngII/AT1R signaling and associated oxidative stress, resulting in a unique state of vascular homeostasis. This supports a broader therapeutic use of this class of medications in a range of vascular diseases and may lead.

## Methods

### Animals

Male wild type (WT) C57BL/6 and eNOS KO mice (Stock 00,664, 002,684) were purchased from The Jackson Laboratory and fed regular 5001 chow diet, whereas fibrillin-1 (FBN1) *C1041G*^+*/−*^. MFS mice were bred in-house with ethical approval from the University of British Columbia Animal Care Committee (UBC ACC) Protocol #A16-0314 and UBC Biosafety Committee #B18-0068. The UBC ACC adheres to Canadian Council on Animal Care (CCAC) and ARRIVE guidelines, with OLAW Assurance #F16-00068 (A5090-01)^15^. 6-week old mice were randomly assigned to receive losartan (0.6 g L^−1^), telmisartan (10 mg kg^−1^), olmesartan (5 mg kg^−1^), valsartan (30 mg kg^−1^), telmisartan + L-NAME (0.5 mg kg^−1^)^15^, captopril (25 mg kg^−1^) or vehicle (drinking water) for either 2 or 18 weeks, until time of sacrifice at 8 or 24 weeks of age, respectively. ARBs were patient formulation-based and purchased at St. Paul’s Hospital pharmacy. Dosages of telmisartan and olmesartan were titrated to give similar BP lowering as a common established dose of losartan (0.6 g L^−1^) used in previous studies^15,18–20^, whereas valsartan was used at a non-BP lowering dose^21^. Drugs in drinking water were replaced three times per week, and dosages were continuously adjusted for body weight and averaged drinking water per day per cage.

### Non-invasive BP measurements and high-resolution ultrasound imaging

Non-invasive BP measurements and ultrasound imaging were performed in mice using tail cuff (CODA 2, Kent Scientific, Torrington, CT) and Vevo2100 systems (MS550D transducer, VisualSonics, FUJIFILM, Toronto, ON, Canada) under very light or moderate isoflurane anesthesia (< 1% or 2% v/v isoflurane at 1.5 L O_2_), respectively. For BP measurements, mice were placed on a warming tray with the tail inserted into an inflatable cuff. After a 10-min acclimation period to minimize variability, BP was measured for 15 cycles and mean, systolic and diastolic BP were calculated using the last 10 cycles. Echocardiograms were performed in a blinded fashion on mice using multiple Sinus of Valsalva measurements (> 5) and averaged^15,22^ at 12 or 24 weeks of age.

### Measurements of isometric force

Mice were sacrificed under terminal isoflurane anesthesia followed by cervical dislocation. The descending thoracic aorta was isolated in ice-cold Krebs solution as previously published with 0.01 mmol L^−1^ ibuprofen to inhibit PGI_2_ synthesis. Aortic segments were mounted on a myograph (AS Danish Myotechnology, Denmark), stretched to the optimal tension (6.0 mN) for 30 min as previously described^23^, followed by two preconstrictions with 30 mmol L^−1^ KCl, then contracted with increasing doses of PE and acetylcholine Ach (0.1 nmol L^−1^ to 100 μmol L^−1^) for contractility and relaxation measurements, respectively. For direct stimulation studies, aortic rings were incubated in the presence of telmisartan (50 μmol L^−1^) or DMSO vehicle in the myograph chambers for 30 min prior to the addition of PE. The dose–response curves were used to calculate maximum force (E_max_) and sensitivity (EC_50_) of PE and Ach. The contribution of NO and AngII receptor type-2 (ATR2) to telmisartan-induced vasodilation was determined by preincubation with L-NAME (200 μmol L^−1^, 30 min)^15^ and the ATR2 blocker PD123319 (30 min; 10 μmol L^−1^) respectively^24^, before addition of PE, whereas NO sensitivity was tested with sodium nitroprusside (0.1 nmol L^−1^ to 100 μmol L^−1^).

### Aortic RNA extraction and gene expression profiling

Mice were mice treated with telmisartan for 3 weeks, their thoracic aortas placed in 400ul of RNAsol (Sigma Aldrich) and homogenized (OMNI International). RNA was extracted after overnight precipitation in isopropanol and resuspended in RNAse free water + 1:20 superase. RNA sample integrity was tested as previously described using the Agilent Bioanalyzer 2100^25^. All computational analyses of RNA-seq data were performed in R (v 3.6.1). Genes with less than 2 counts per million (CPMs) in at least two samples were removed. Batch correction was applied using Combat-seq under the sva package (v. 3.35.2) with genotype and treatment specified as biological covariates^26^. Batch-adjusted data were processed and analyzed using DESeq2 (v 1.24.0)^27^ with a design formula accounting for genotype, treatment, and their interaction including normalization, principal component analysis (PCA), and Wald test differential expression analysis. Heatmaps represented regularized log-transformed gene expression normalized by Z-score. Pathway enrichment analysis (Gene Ontology GO) was performed using clusterProfiler (v 3.12.0)^28^. Log-transformed p-values and the number of genes mapped to each annotation are depicted on the dot plot.

### Histology

Bisected parallel to the atrioventricular plane, paraffin-embedded hearts were cut throughout the aortic root at the Sinus of Valsalva as previously published^15^, stained with Verhoeff-van Geison (VVG). Elastic fiber fragmentation was assessed using ImageJ software by tracing individual fibers and measuring their length in pixels followed by conversion into micrometers^29^. Medial thickening was determined using Aperio ImageScope software (Leica Biosystems, Wetzlar, Germany) by measuring the average distance between 4 internal and external elastin lamina images. For immunohistochemistry, sections were subjected to antigen retrieval (10 mM citrate buffer, pH 6), endogenous peroxidase quenching, blocked for 2 h with 10% normal goat serum plus 1% BSA and probed for phospho-p44/42 MAPK (ERK 1/2) (Cell Signaling Technology, Cat# 9101S) using DAB (Vector Laboratories) and hematoxylin counterstain.

### Western blot

Unfixed frozen hearts from WT mice treated with telmisartan for two weeks were homogenized in lysis buffer and centrifuged^16^. Following protein quantification, samples were resolved using SDS-PAGE and transferred onto nitrocellulose membrane. The membrane was cut just below 100kDs and the top and bottom sections were blocked and probed overnight with primary antibodies for eNOS (1:1000, Cell Signaling, Cat# 32027) on the top membrane, and T-AKT (1:1000, Cell Signaling, Cat# 2920), p-AKT (1:1000, Cell Signaling, Cat# 4060), Cav-1 (1:1000, Santa Cruz Biotechnology, Cat# sc-894) and GAPDH (1:4000, Cell Signaling, Cat# 2118) on the bottom membrane, followed by incubation with goat anti-rabbit AlexaFluor700 (1:5000, Thermo Fisher Scientific, Cat# A-21038) and goat anti-mouse AlexaFluor800 (1:5000, Thermo Fisher Scientific, Cat# A-32730) secondary antibodies before imaging with a Li-COR scanner. The Li-COR software was used to designated the area of interest to be scanned for the bottom membrane.

### Statistical analysis

The data and statistical analysis comply with the recommendations on experimental design and analysis in pharmacology^30^. Comparisons of parameters between only two groups were performed by two-tailed Student’s *t*-test whereas one- or two-way ANOVA was used to compare multiple groups with Bonferroni’s Post-hoc method for multiple comparisons if the necessary level of statistical significance was reached using GraphPad Prism 6.01 (San Diego, CA, USA). Data are presented as the means ± SEM, with a *p* < 0.05 considered significant. For gene expression data, p-values were subjected to Benjamini–Hochberg correction and only significant results were considered in differential expression and pathway enrichment analysis.

## Results

### Chronic ARB treatment enhances eNOS-dependent endothelial function

**Figure 1:**
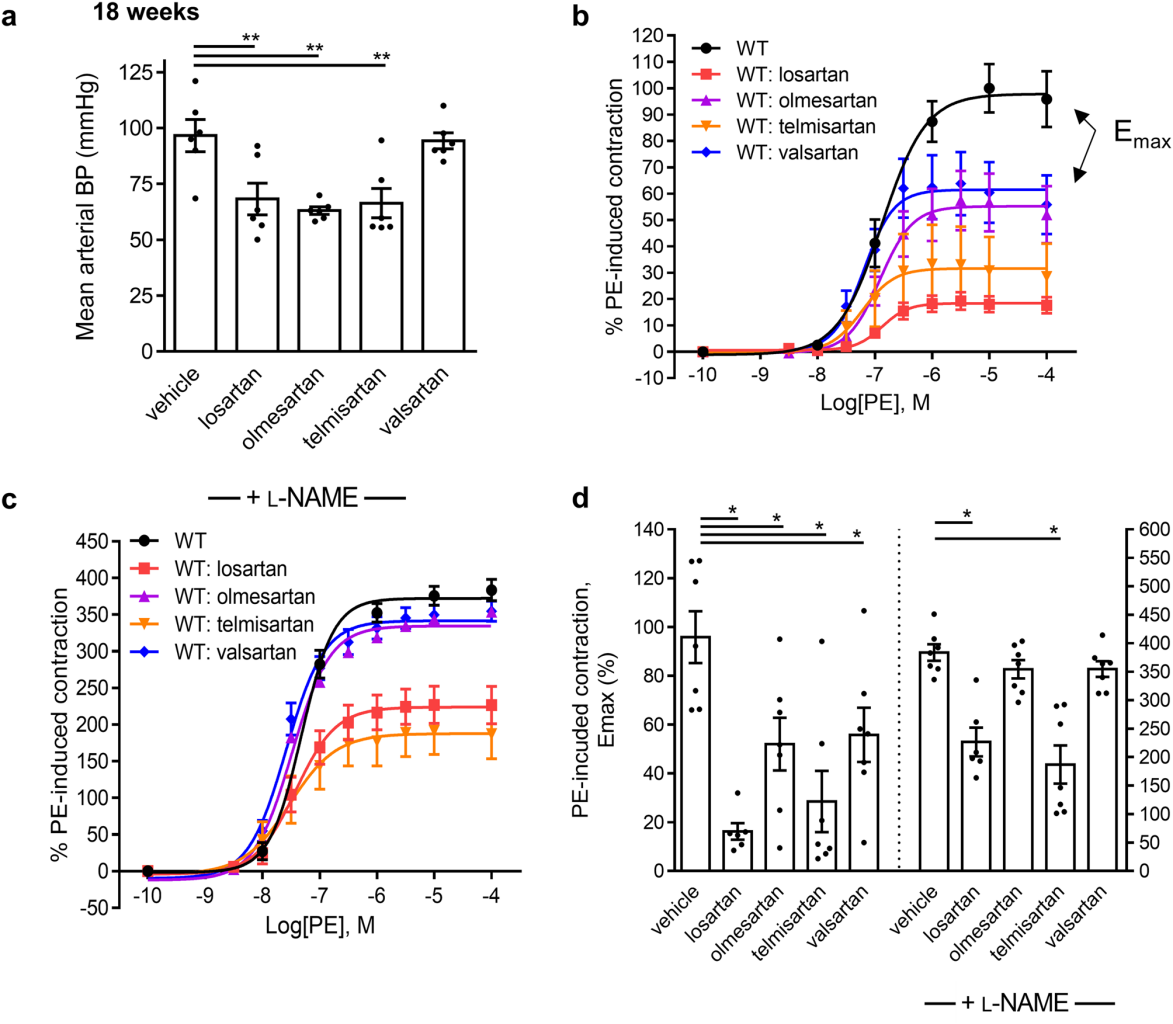
Long term treatment with ARBs reduce aortic vessel contractility in a NO-dependent manner. (**a**) Mean arterial BP of mice after long-term treatment with ARBs for 18 weeks. (**b**) Long term administration of four different ARBs reduces PE-induced contraction of mouse aorta. (**c**) Pre-treatment with the NOS inhibitor L-NAME increases PE-induced force fourfold in the controls and abolishes the ARB-induced decrease in force development. (**d**) Maximum force (E_max_) values in the absence and presence of L-NAME of aorta from control and ARB treated mice. One experimental mouse was euthanized due to malocclusion according to standard ethics protocols. (vehicle n = 7, losartan n = 6, olmesartan n = 7, telmisartan n = 7, valsartan n = 7).

To determine if endothelial function activation is a shared ARB class-effect, WT mice were treated for 18 weeks with either losartan, olmesartan, telmisartan or valsartan. ARB treatments were well-tolerated and resulted in no changes in body weight (Supplementary Fig. S1). As expected, lowering of BP varied between medications (Fig. 1a). Aortic ring myography revealed that all 4 ARBs investigated induced significant 82, 48, 72, and 45% decreases in maximum PE (0.1 nmol L^−1^ to 100 μmol L^−1^)-induced contraction (E_max_), respectively (Fig. 1b). As expected, ARBs drastically reduced PE-induced preconstruction prior to Ach stimulation and this resulted in inconsistent endothelium-dependent vasodilation (data not shown) as previously reported^31^. NOS inhibitor L-NAME rescued the contractions of both treated and untreated groups (Fig. 1b-d), confirming a chronic NOS-dependent up-regulation of endothelial function by ARBs.

**Figure 2:**
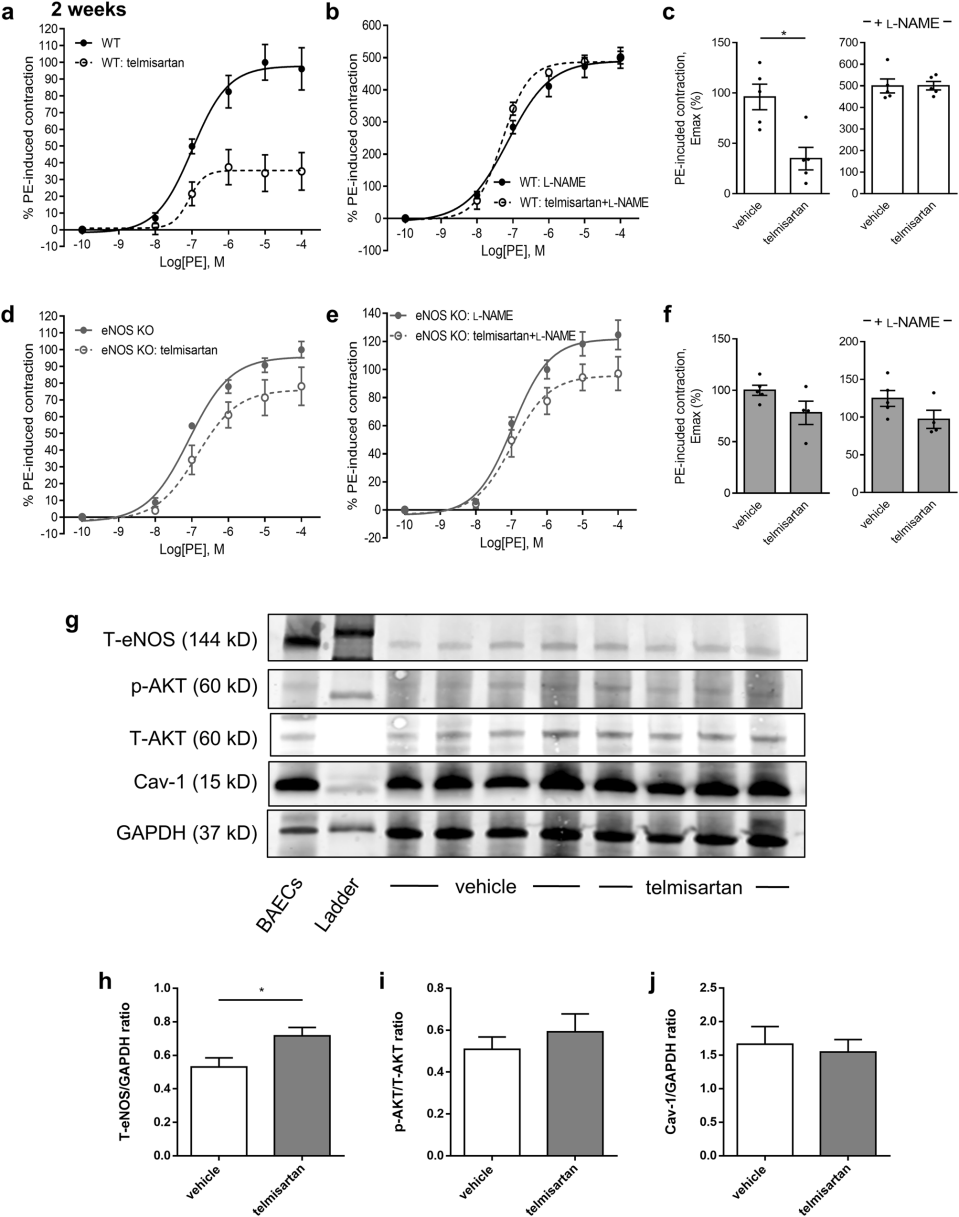
eNOS KO tissues are resistant to telmisartan. (**a**) Short-term two-week treatment with telmisartan results in reduced PE-induced contraction of WT mouse aorta. (**b**) Pre-treatment with the NOS inhibitor L-NAME increases PE-induced force in the controls and abolishes the ARB-induced decrease in force development. (**c**) E_max_ values in the absence and presence of L-NAME of aorta from WT mice treated with telmisartan. (**d**-**e**) Loss of aortic contractility in (**A**) is abrogated in eNOS KO mice. (**f**) E_max_ values in the absence and presence of L-NAME of aorta from eNOS KO mice treated with telmisartan. (**g**-**j**) Western blots of eNOS and associated co-factors performed in hearts from WT mice treated with telmisartan for 2 weeks. (n = 5 for all groups).

Two-week WT mouse treatment with telmisartan, a pleotropic ARB with one of the greatest reductions on PE E_max_, reduced PE contractility by 63% in a fully L-NAME-sensitive manner (Fig. 2a-c) but did not significantly reduce aortic contractility (Fig. 2d) in eNOS-null mice (Fig. 2e-f). Combined with data showing similar treatment with ACEi captopril at a similar BP-lowering dose as telmisartan resulted in no significant endothelial function activation (Supplementary Fig. S2), these experiments suggest that long-term ARB activation of eNOS-derived vasodilatory NO may be independent of AngII inhibition and separate from the BP-lowering properties of ARBs. Telmisartan did not significantly modulate endothelium-independent vasodilation but enhanced the contractile response to L-NAME even in absence of PE (Supplementary Fig. S3). Western blot analyses revealed a significant increase of total eNOS protein levels, but not of phospho-AKT or Cav-1, in WT cardiac tissues following a 2-week telmisartan treatment, lending credence to observations of increased eNOS-derived NO vasodilation in ARB-treated mice (Fig. 2g-j, Supplementary Fig. S4).

### Direct ex vivo stimulation of aortic segments with telmisartan increases endothelium-dependent vasodilation in absence of AngII or ATR activity

**Figure 3:**
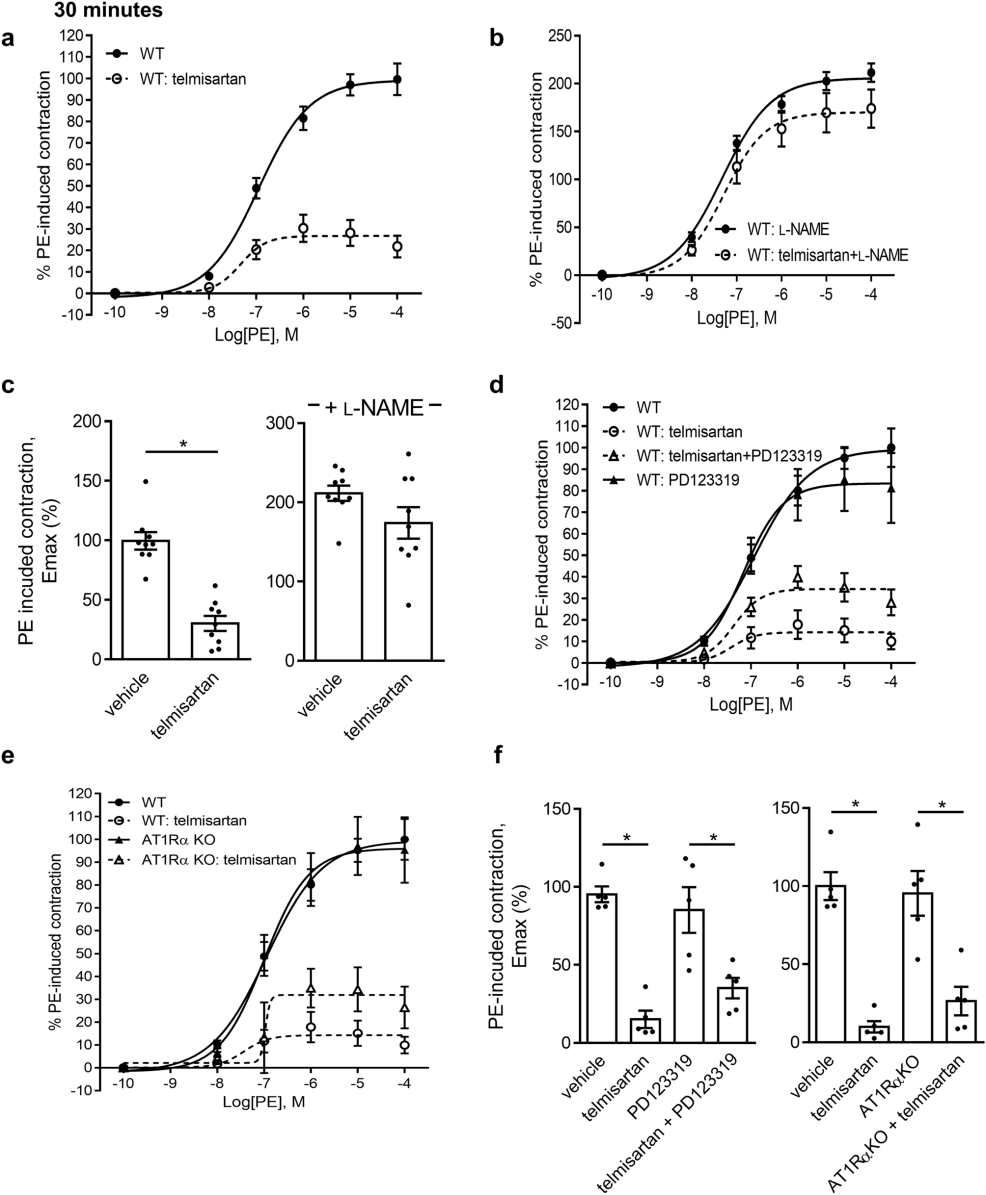
Direct telmisartan stimulation results in reduced NO-dependent aortic contractility. (**a**) Direct ex vivo addition of telmisartan (30 min; 50 μmol L^−1^) reduces PE-induced aortic contraction in the aorta of WT mice. (**b**) Pre-treatment with the NOS inhibitor L-NAME increases PE-induced force in the controls and abolishes the ARB-induced decrease in force development. (**c**) E_max_ values of telmisartan-treated aortic segments in the absence and presence of L-NAME. (WT n = 9, telmisartan n = 9). (**d**) Effect of direct ex vivo addition of PD123319 (30 min; 10 μmol L^−1^) on telmisartan-induced aortic contractility. (**e**) Direct ex vivo addition of telmisartan (30 min; 50 μmol L^−1^) reduces PE-induced aortic contraction in the aorta of WT and AT1Rα KO mice. (**f**) E_max_ values of direct telmisartan co-treatment with PD123319 and in AT1Rα KO mice. (n = 5 for all groups).

We observed a significant 70% reduction in PE-induced aortic contractility following a 30-min treatment with telmisartan (50 μmol L^−1^) as compared to DMSO-treated vessels in absence of AngII, an effect not observed in the presence of L-NAME (Fig. 3a-c). No differences were observed in Ach-induced relaxation (Supplementary Fig. S5a-c). As AT1R antagonism by ARBs may cause a switch towards protective ATR2 signaling in presence of AngII, we tested the ATR2 blocker PD123319 on the acute NO-enhancing properties of telmisartan, and observed no significant inhibition of telmisartan-induced vasodilation independently of L-NAME (Fig. 3d,f, and SIII). Use of AT1Rα KO aortas did not interfere with telmisartan-induced vasodilation, further supporting possible AngII/ATR pathway-independent effects of telmisartan (Fig. 3e-f, and Supplementary Fig. S5). We conclude that telmisartan is capable of rapid pleiotropic stimulation of eNOS in the absence of exogenous AngII and independently of the classic AngII/ATR system.

### Telmisartan protects against age- and MFS-associated aortic root pathology in an endothelial function-dependent manner

**Figure 4:**
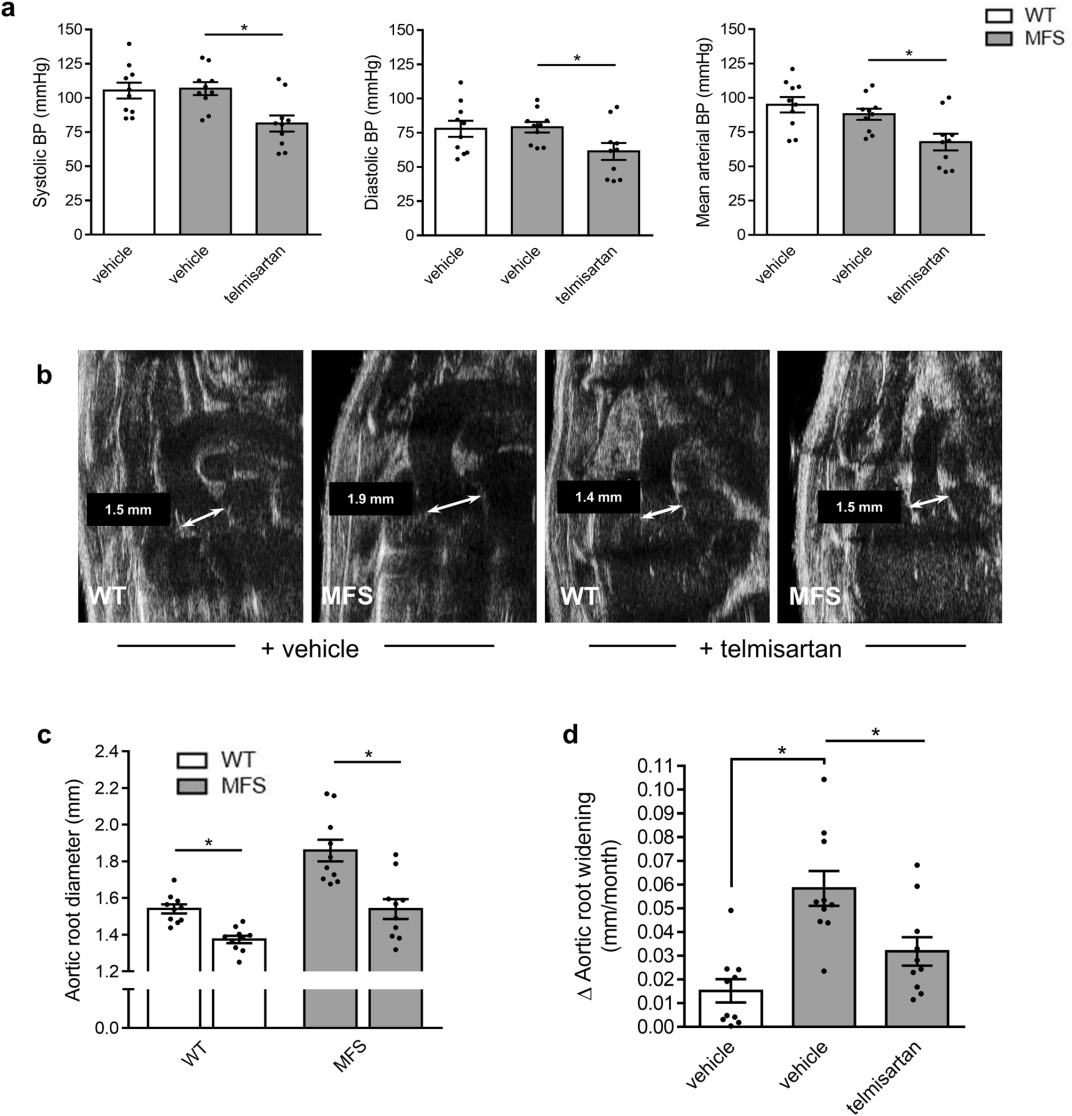
Telmisartan attenuates aortic root widening in MFS mice. (**a**) Long-term treatment with telmisartan results in reduced BP in MFS mice. (**b**) Representative echocardiograms of aortic roots of WT controls and MFS mice treated long-term with telmisartan. (**c**) Telmisartan treatment results in reduced aortic root diameter in MFS mice and (**d**) a lower rate of aortic root widening in MFS mice compared to vehicle treated MFS mice. (WT n = 10, MFS n = 10, MFS + telmisartan n = 10).

**Figure 5:**
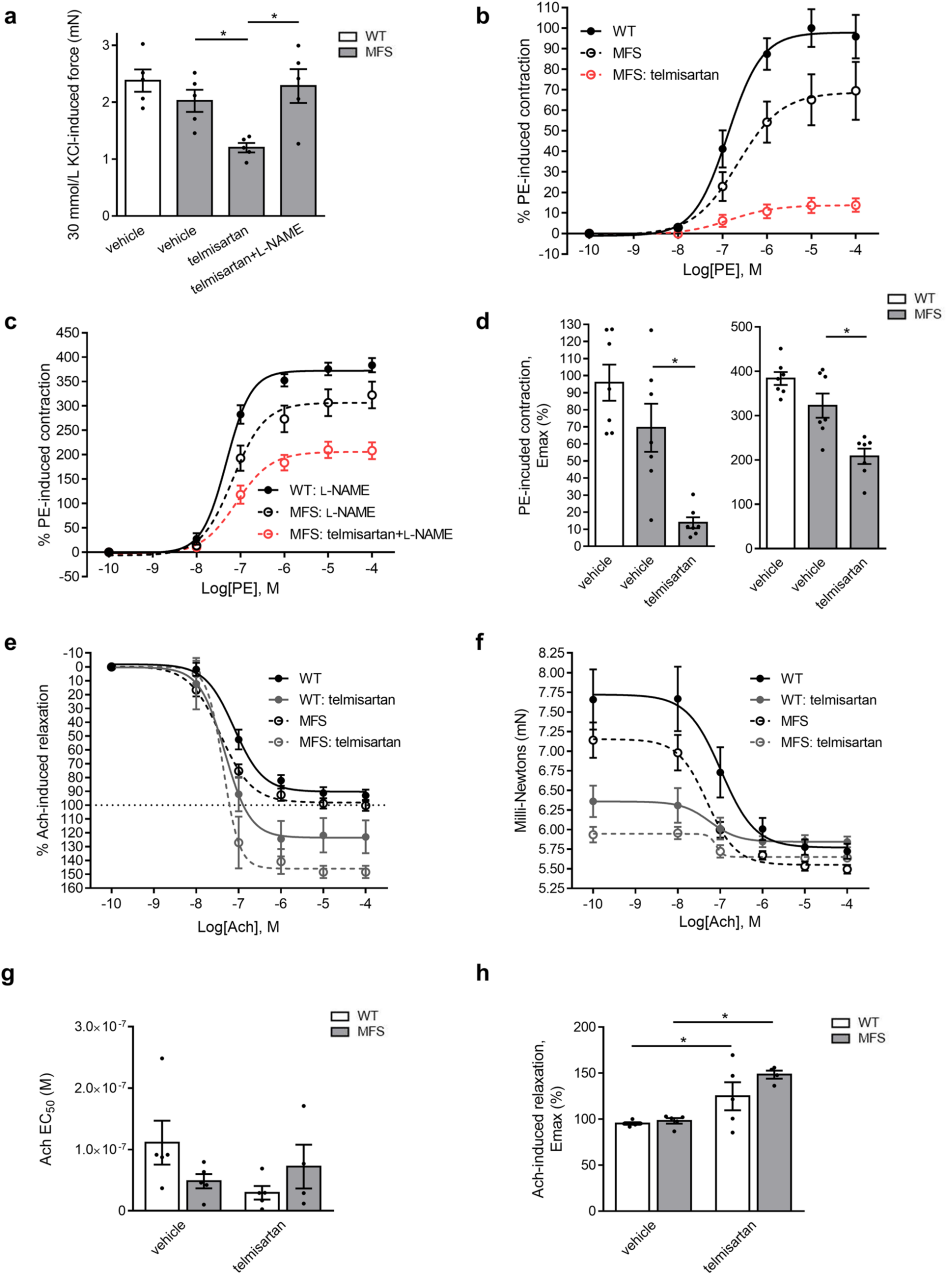
Telmisartan treatment leads to enhanced endothelial function in the aorta of MFS mice. (**a**) Agonist independent high K^+^-induced contraction is reduced in the aortic vessels of MFS mice treated with telmisartan. Pre-treatment with the NOS inhibitor L-NAME abolishes the ARB-induced decrease in force development (n = 5 for all groups). (**b**) Long term administration of telmisartan reduces PE-induced contraction of MFS mouse aorta. (**c**) Pre-treatment with the NOS inhibitor L-NAME increases PE-induced in the controls and abolishes the ARB-induced decrease in force development. (**d**) E_max_ values in the absence and presence of L-NAME of WT and MFS aorta from vehicle and telmisartan treated mice (n = 7 for all groups). (**e**–**f**) Long term administration of telmisartan increases Ach-induced relaxation of MFS mouse aorta. Ach-induced relaxation (**g**) E_max_ and (**h**) EC_50_ values of MFS mice aorta treated with telmisartan (n = 5 for all groups).

**Figure 6:**
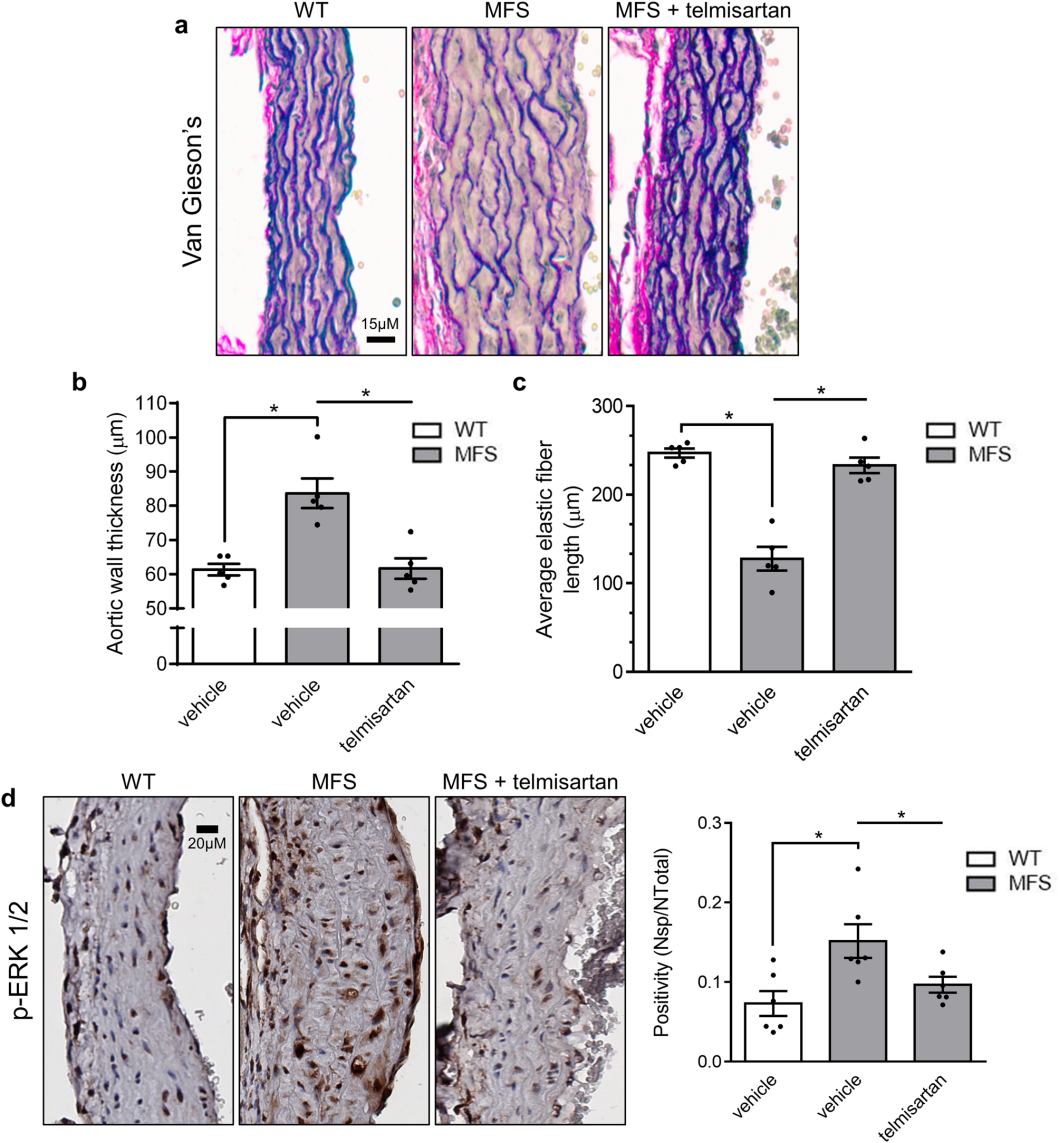
Telmisartan attenuates MFS-associated pathological remodeling and signalling in the aortic root of MFS mice. (**a**) Representative Van Geison’s staining of aortic roots of WT and MFS mice treated long-term with telmisartan. Treatment with telmisartan (**b**) decreases aortic wall thickness and (**c**) increases average length of elastic fibers in the aortic root of MFS mice. (WT n = 5, MFS n = 5, MFS + telmisartan n = 5) (**d**) Representative p-ERK ½ staining, and average quantification of aortic roots of WT and MFS mice treated long-term with telmisartan. (WT n = 6, MFS n = 6, MFS + telmisartan n = 6).

The therapeutic relevance of endothelial function enhancement by telmisartan was tested in aging- and MFS-accelerated aortic root widening and remodeling. As observed with WT mice (Fig. 1), telmisartan reduced mean arterial BP of MFS mice by 22 mmHg (Fig. 4a). Echocardiography measurements at 24 weeks revealed that MFS-associated aortic root widening was completely prevented by telmisartan treatment (Fig. 4b,c, grey bars), bringing the rate of aortic root widening close to healthy controls (Fig. 4d). The anti-aortic root remodeling effect of telmisartan was such that even age-related aortic root widening in WT mice was significantly reduced compared to vehicle controls (Fig. 4c, white bars). Wire myography confirmed that telmisartan attenuated agonist-independent KCl contractility of MFS aortic rings in a fully L-NAME-sensitive fashion (Fig. 5a). Compared to untreated MFS animals, telmisartan lowered PE-induced MFS contractility by 82% (Fig. 5b), an unexpected observation considering reports of inducible NOS expression in MFS aortas, whereas L-NAME normalized untreated WT and MFS vessels and greatly potentiated the contractility of telmisartan treated vessels, although not as much as the WTs (Fig. 5c-d). Acetylcholine (Ach)-induced endothelium-dependent relaxation was significantly increased to unusually high levels in MFS mouse aortas following long-term treatment with telmisartan (Fig. 5e-h), which could be attributable to very weak pre-constriction. This correlated with complete inhibition of MFS aortic root pathology, as medial thickening (Fig. 6a, b) and elastic fiber fragmentation (Fig. 6c) observed in MFS aortas returned to naïve WT levels. Mechanistically, telmisartan attenuated the pathological phosphorylation of extracellular-regulated kinase (ERK) 1/2 at 24 weeks of age (Fig. 6d), a well-established marker of non-canonical TGF-β signaling^18^.

**Figure 7:**
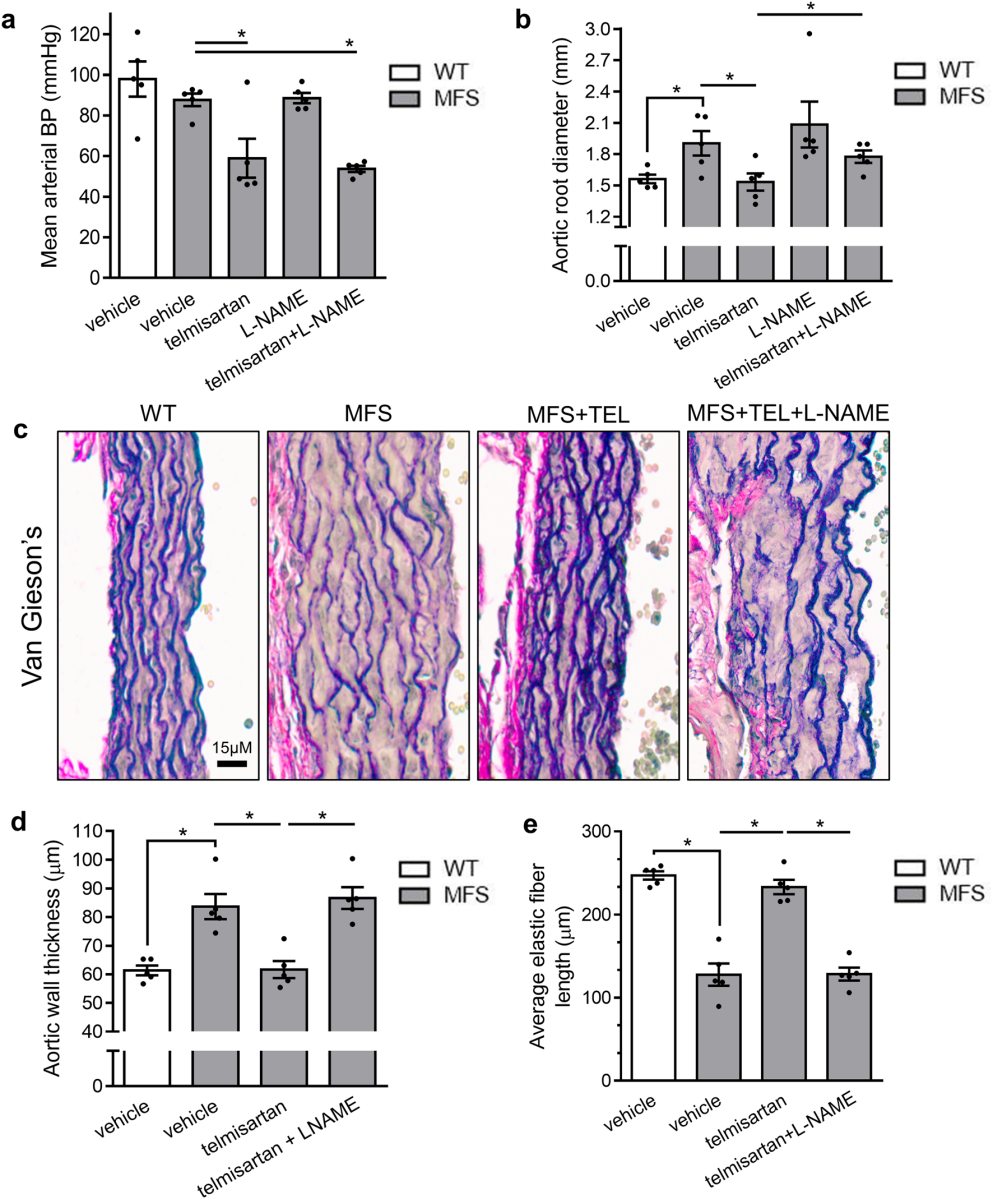
Telmisartan (TEL) inhibits MFS-associated aortic root pathology in a NOS-sensitive fashion. (**a**) Long-term treatment with telmisartan results in reduced BP in MFS mice, an effect that is sustained during co-treatment of telmisartan with L-NAME. (**b**) Telmisartan loses its anti-aortic root widening effects following long-term co-treatment of telmisartan with L-NAME in MFS mice. (**c**) Representative Van Geison’s staining of aortic roots of WT and MFS mice treated with telmisartan and/or L-NAME. Co-treatment with telmisartan and L-NAME abolishes telmisartan’s effects on (**d**) decreasing aortic wall thickness and (**e**) increasing elastic fiber length. (n = 5 for all groups).

Causality between telmisartan’s endothelial function-activating effects and its anti-aortic root remodeling properties was further investigated with L-NAME. In vivo NOS inhibition in combination with telmisartan did not alter telmisartan’s BP-lowering effects (Fig. 7a) in MFS mice but attenuated its anti-aortic root widening effects by 63% when compared to telmisartan alone (Fig. 7b) and completely blocked its protective effects on aortic medial thickening and elastic fiber fragmentation (Fig. 7c-e). Thus, we conclude that endothelial NO release, rather than BP lowering, is critical to the anti-aortic root remodeling properties of telmisartan.

### Telmisartan triggers eNOS-dependent aortic transcriptome reprogramming

**Figure 8:**
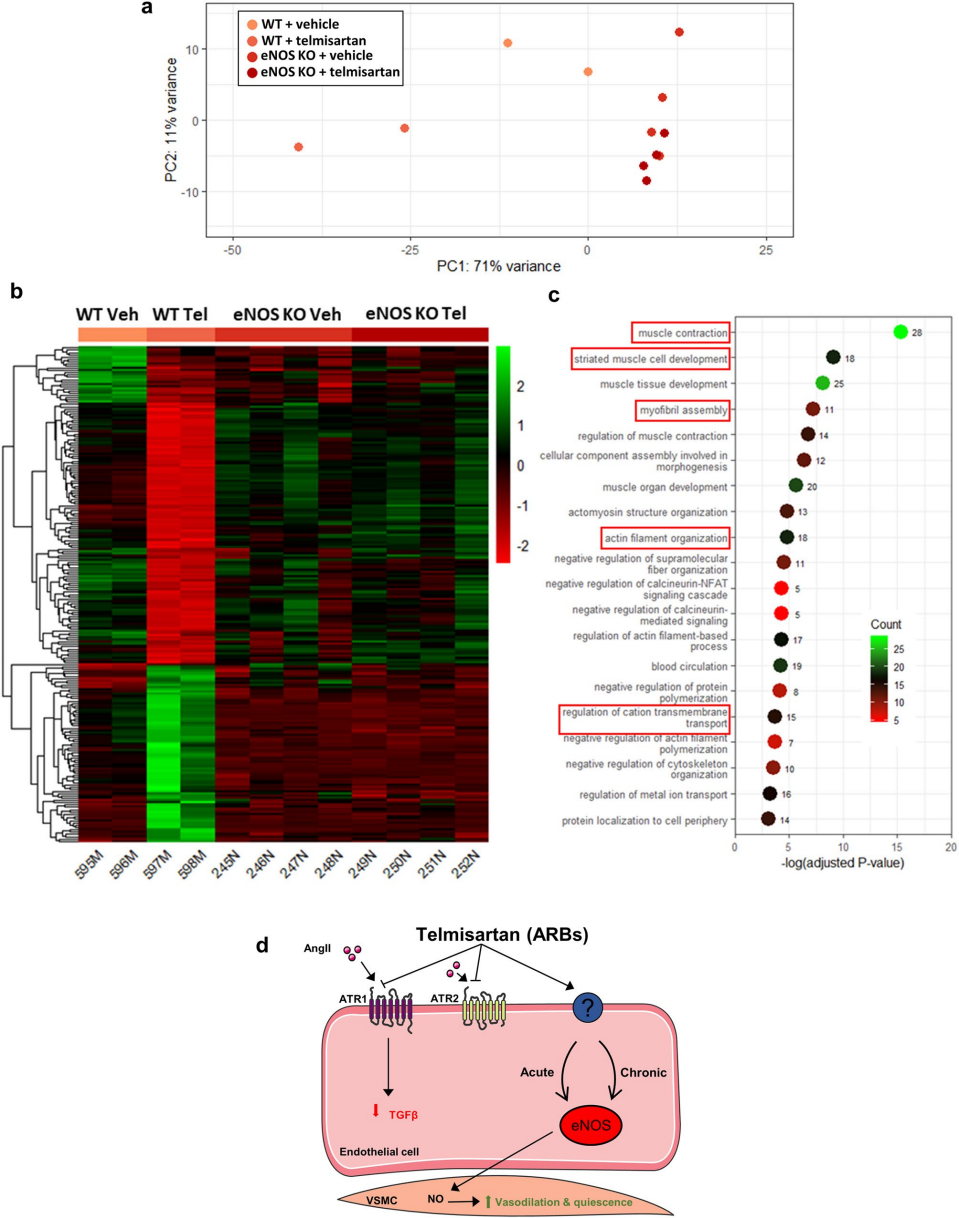
Effect of telmisartan on eNOS-dependent gene signature changes by RNA-seq. (**a**) Principle component analysis (PCA) of the transcripts detected in aortic tissues isolated from WT and eNOS KO mice. (**b**) Expression clustering of samples and regions significantly different between untreated and treated WTs but not significantly different between untreated and treated eNOS KO mice. (**c**) Top 20 gene ontology (GO) terms with the number of associated DEGs related to eNOS-dependent pathways regulated by telmisartan in the mouse aorta. (WT n = 2, WT + telmisartan n = 2, eNOS KO n = 4, eNOS KO + telmisartan n = 4). (**d**) Mechanistic overview of how ARBs/telmisartan act on eNOS and how this goes on to also have protective effects on vascular remodeling.

To better understand how telmisartan-induced eNOS-derived endothelial function activation prevents aortic root remodeling, bulk RNA-seq analyses were performed on aortic tissues from WT and eNOS KO mice treated with vehicle or telmisartan for 3 weeks. Transcriptome principle component analysis (PCA) revealed distinct expression profiles between vehicle- and telmisartan-treated WT samples, whereas eNOS KO samples showed minimal variation regardless of treatment (Fig. 8a). Telmisartan induced early transcriptomic changes in the aorta associated with increased expression of genes involved in mitochondrial and metabolic processes and decreased expression of genes involved in ECM organization, connective tissue development, and response to TGF-β (among others) (Supplementary Fig. S6). Telmisartan treatment was associated with 2301 differentially expressed genes (DEGs; up: 1157 down: 1144) in WT aorta, including attenuation of TGF-β-related genes (TGFB2, 1.6-fold; Smad7, 1.4-fold; latent TGF-β binding protein 1, 1.33-fold). On the other hand, telmisartan treatment was associated with only 260 DEGs in KO aortas. We found 261 genes that showed an interaction effect between genotype and treatment and of these 261 genes, 219 were differentially expressed in WT mice (up: 79 down: 140) and not in eNOS KO mice following telmisartan treatment (Fig. 8b). GO biological pathway analysis of these genes revealed annotations related to muscle contraction and development, myofibril assembly, and actin filament organization (Fig. 8c). Together, theses results suggest that eNOS plays an important role in the regulation of the aortic transcriptome following telmisartan treatment (Fig. 8d).

## Discussion

In this study, we provide evidence that chronic administration of ARBs in mice results in profound reduction of vascular tone through activation of eNOS-derived NO, even at sub-BP lowering doses, something not observed following similar ACE inhibition or in eNOS-null animals. Acute ex vivo treatment of blood vessels with telmisartan, a pleiotropic ARB with AT1R-independent signaling properties, also causes eNOS-dependent inhibition of aortic contractility in fashions not linked to classic AngII/AT1R signaling. Telmisartan protection against age- and MFS-associated aortic root pathology in mice requires an intact NO system and results in eNOS-specific changes to the aortic transcriptome. This sheds new light on the unrecognized role of endothelial function activation as a critical mediator of telmisartan’s therapeutic properties and may support its broader prophylactic use to prevent vascular disease, particularly in MFS patients as management with gold-standard losartan leads to underwhelming aortic root stability.

The pathways by which ARBs can activate basal NO-dependent endothelial function both in vivo and ex vivo is unknown. While the AngII/AT1R system is one of the main regulators of BP, it is also a potent activator of oxidative stress, particularly that of xanthine and NADPH oxidases. However, ARBs, even when used at sub-BP doses in the case of valsartan, were capable of profound, chronic endothelial function activation whereas captopril used at a similar BP lowering dose as telmisartan caused little endothelial function activation, arguing against an AngII-dependent effect. Inhibition of ACE activity can also enhance levels of potent vasodilator bradykinin^32^, but also reduce protective AngII/AT2R signaling^33^ whereas AT1R blockade can shift AngII signaling towards vasodilatory AT2R activity^34^. However acute inhibition of vascular tone with telmisartan in absence of AngII ex vivo does not support an AT2R-dependent signaling switch, which requires AngII.

As others have shown how telmisartan can activate peroxisome proliferator-activated receptor-γ in absence of AngII and losartan metabolism can generate biologically active molecules lacking AT1R-blocking capabilities^35,36^, it is tempting to assume that ARBs activate endothelial function independently of AT1R. To err on the side of caution, ARBs can act on the AT1R system in fashions far more complex than typical antagonists; candesartan was depicted as an inverse agonist due to its capacity to block mechanical stress-induced AT1R stimulation in absence of AngII^37^. This ARB was also shown to trigger AngII-independent production of inositol phosphate via a unique set of interactions with AT1R that differ from AngII antagonism whereas site-directed mutagenesis of AT1R has identified the critical residues required for such inverse agonist action^38^. AT1R has also been shown to signal as a monomer, homodimer and heterodimer with other G-protein coupled receptors including AT2R, which further convolutes the true mechanism of action of ARBs on vascular tone. Future studies are required to fully elucidate the endothelial target of ARBs and optimize the drug-receptor interactions involved.

An unexpected outcome of our study is the characterization of telmisartan’s robust anti-aortic root remodeling, both in aged and MFS mice. β-blocker atenolol^39^ and ARBs, particularly losartan, are still guideline-recommended for management of MFS-associated aortic root disease^40^, but whether other ARBs such as telmisartan could offer superior prophylactic protection is an interesting possibility. ACEi enalapril was shown to be inferior to losartan at abrogating MFS-associated aortic aneurysm, casting doubts about the true contribution of AngII signaling in this disease. In contrast, moderate aerobic exercise, the ‘gold standard’ endothelial function activator, provides protection against aortic root widening in MFS^41^. Recent investigations have shown that changes in mean arterial BP or systolic BP did not correlate with aortic root dilatation rates in usually normotensive MFS patients treated with losartan or controls^42^, which supports our previous findings that the BP lowering effects of ARBs may be of low significance in MFS^15^. The eNOS-specific changes in differential gene expression we observed in the aorta following only 3 weeks of telmisartan treatment suggest that endothelial function activation by ARBs regulate many genes relating to SMCs function and contraction, which likely influence the phenotypic switch of medial SMCs between a generally healthy, contractile phenotype to a more synthetic and pathologic phenotype often observed in cardiovascular disease. Interestingly, the most significant eNOS-dependent DEGs in the aorta following telmisartan treatment was upregulation of the long non-coding RNA H19 which has recently been shown to exert protective effects on the endothelium via eNOS activation^43^. These novel insights into the pleiotropic actions of ARBs will likely guide future research into the mechanism of ARB-induced activation of eNOS and ARB-endothelial receptor interactions, with the ultimate aim of improving drug therapy of vascular disease.

## Supplementary Information

Supplementary Information.

## Data Availability

The datasets generated and/or analysed during the current study are not publicly available due to current ongoing continuation studies but are available from the corresponding author on reasonable request.
