# Supplementary material for: Pleiotropic activation of endothelial function by angiotensin II receptor blockers is crucial to their protective anti-vascular remodeling effects
Source: Sci Rep. 2022 Jun 13;12:9771. doi: 10.1038/s41598-022-13772-3 (PMC9192586; doi:10.1038/s41598-022-13772-3)
Supplement: Supplementary file 1 — Supplementary Information. [file 41598_2022_13772_MOESM1_ESM.pptx]

## Slide 1
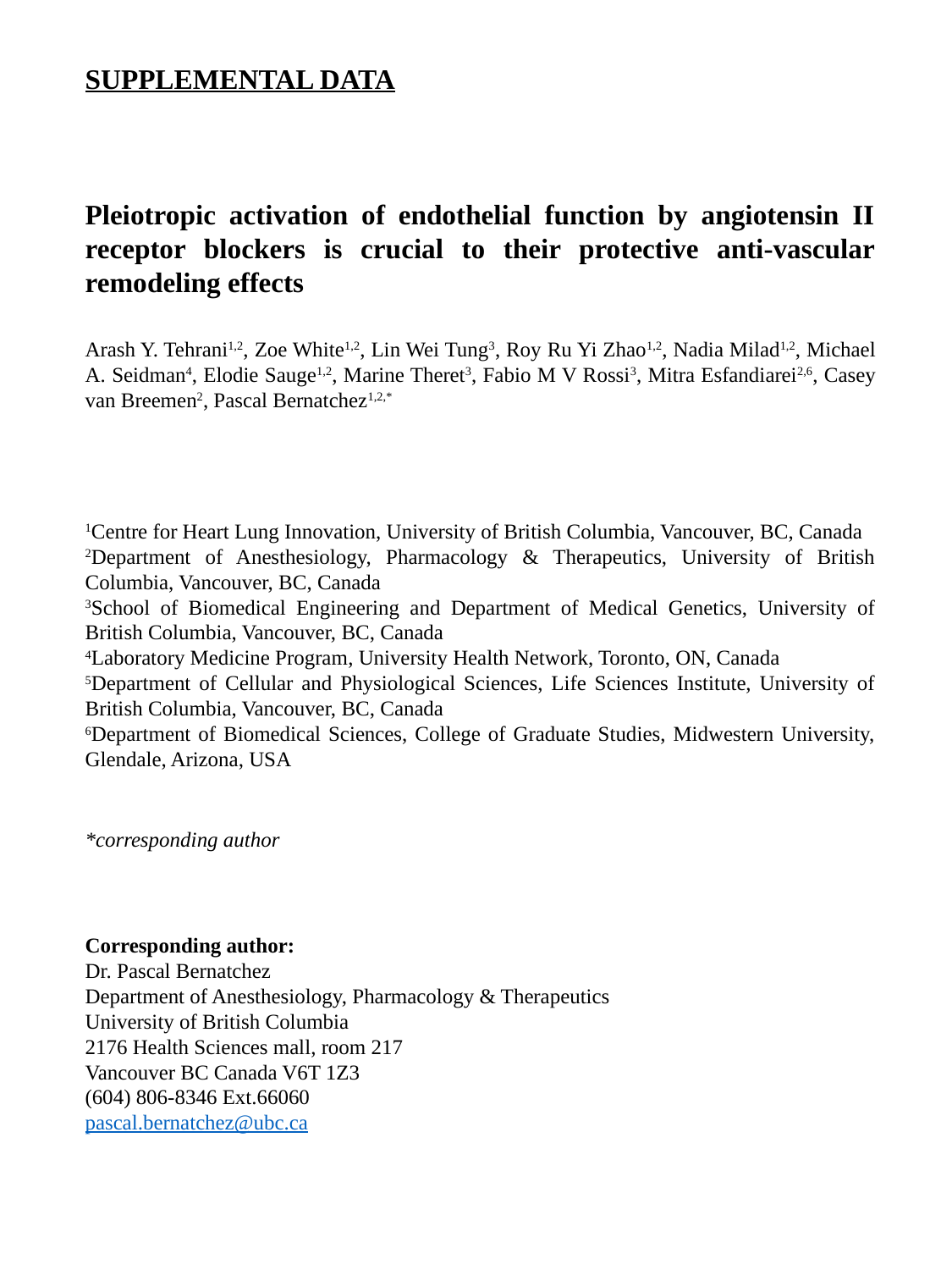

SUPPLEMENTAL DATA
Pleiotropic activation of endothelial function by angiotensin II receptor blockers is crucial to their protective anti-vascular remodeling effects
Arash Y. Tehrani1,2, Zoe White1,2, Lin Wei Tung3, Roy Ru Yi Zhao1,2, Nadia Milad1,2, Michael A. Seidman4, Elodie Sauge1,2, Marine Theret3, Fabio M V Rossi3, Mitra Esfandiarei2,6, Casey van Breemen2, Pascal Bernatchez1,2,*
1Centre for Heart Lung Innovation, University of British Columbia, Vancouver, BC, Canada
2Department of Anesthesiology, Pharmacology & Therapeutics, University of British Columbia, Vancouver, BC, Canada
3School of Biomedical Engineering and Department of Medical Genetics, University of British Columbia, Vancouver, BC, Canada
4Laboratory Medicine Program, University Health Network, Toronto, ON, Canada
5Department of Cellular and Physiological Sciences, Life Sciences Institute, University of British Columbia, Vancouver, BC, Canada
6Department of Biomedical Sciences, College of Graduate Studies, Midwestern University, Glendale, Arizona, USA
*corresponding author
Corresponding author:
Dr. Pascal Bernatchez
Department of Anesthesiology, Pharmacology & Therapeutics
University of British Columbia
2176 Health Sciences mall, room 217
Vancouver BC Canada V6T 1Z3
(604) 806-8346 Ext.66060
pascal.bernatchez@ubc.ca

## Slide 2
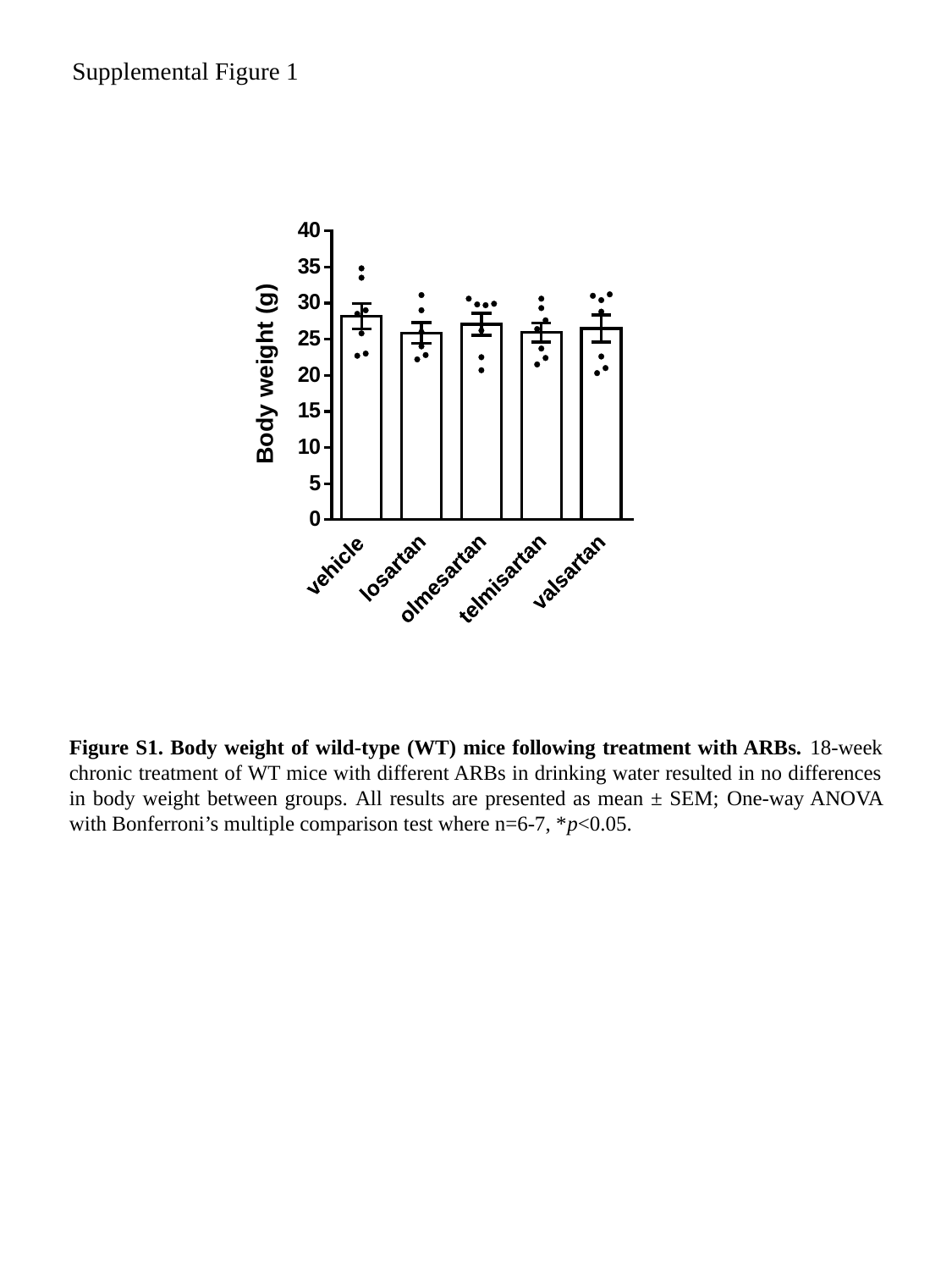

Supplemental Figure 1
Figure S1. Body weight of wild-type (WT) mice following treatment with ARBs. 18-week chronic treatment of WT mice with different ARBs in drinking water resulted in no differences in body weight between groups. All results are presented as mean ± SEM; One-way ANOVA with Bonferroni’s multiple comparison test where n=6-7, *p<0.05.

## Slide 3
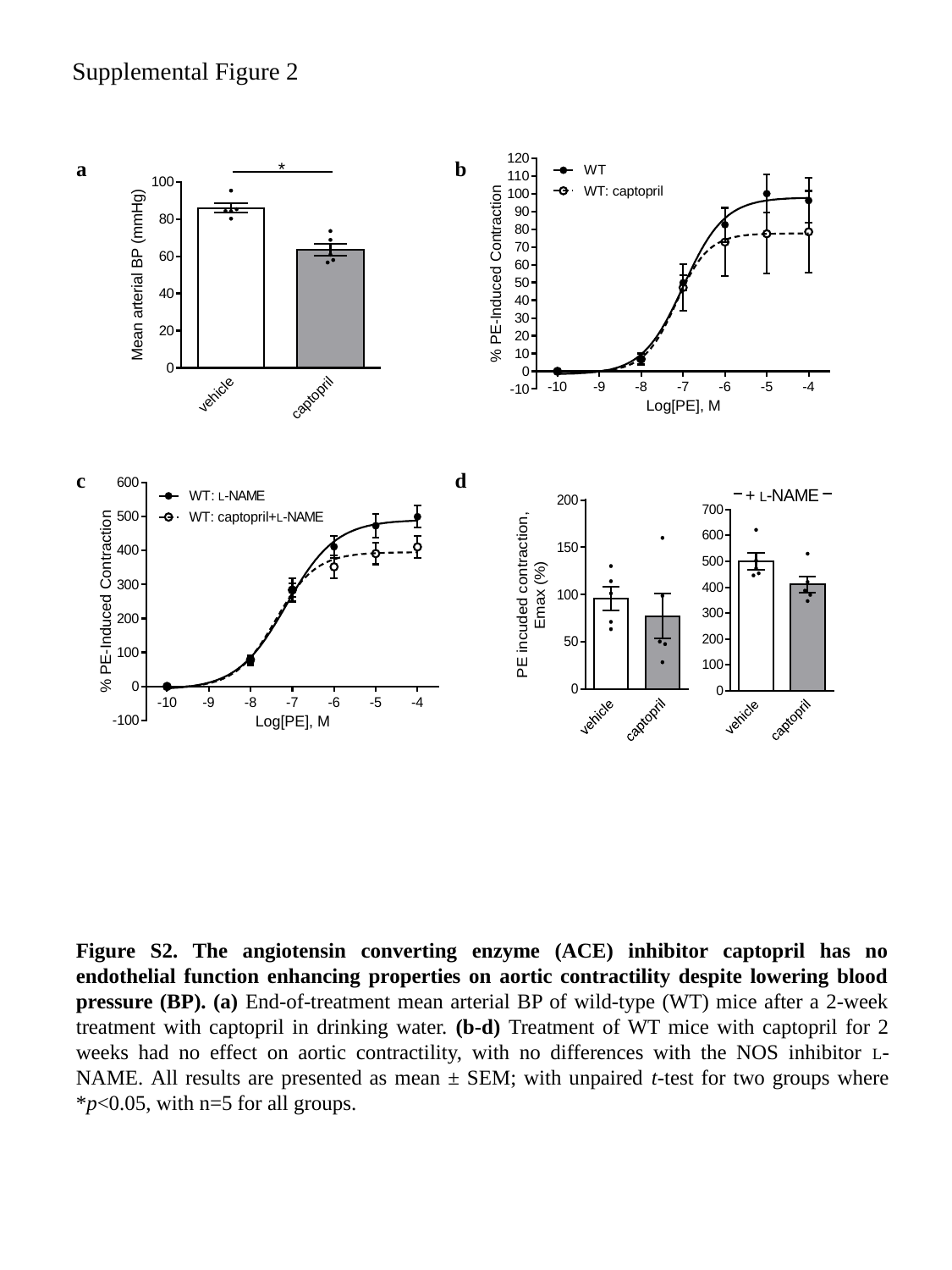

Supplemental Figure 2
a
b
c
d
Figure S2. The angiotensin converting enzyme (ACE) inhibitor captopril has no endothelial function enhancing properties on aortic contractility despite lowering blood pressure (BP). (a) End-of-treatment mean arterial BP of wild-type (WT) mice after a 2-week treatment with captopril in drinking water. (b-d) Treatment of WT mice with captopril for 2 weeks had no effect on aortic contractility, with no differences with the NOS inhibitor L-NAME. All results are presented as mean ± SEM; with unpaired t-test for two groups where *p<0.05, with n=5 for all groups.

## Slide 4
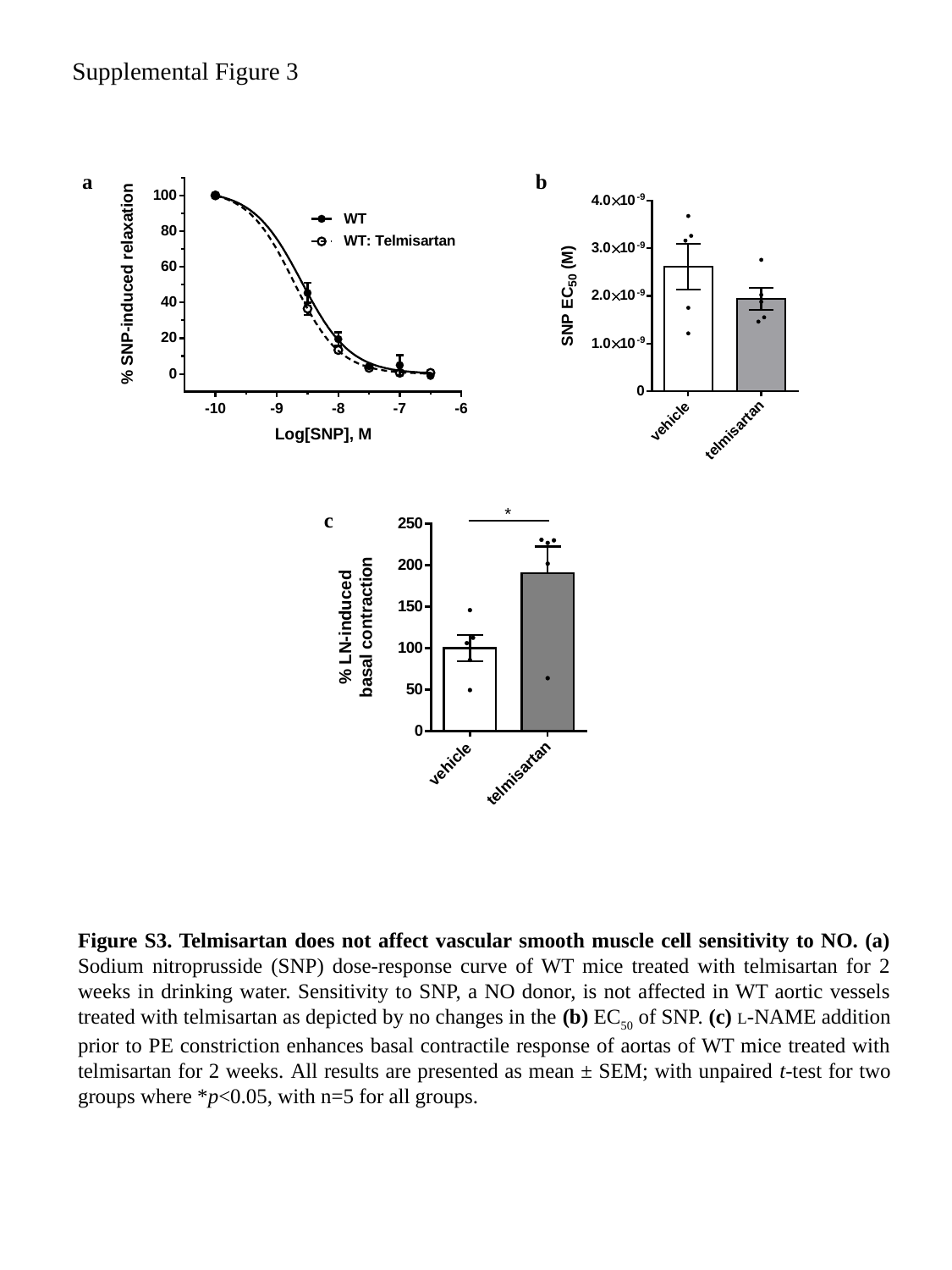

Supplemental Figure 3
a
b
c
Figure S3. Telmisartan does not affect vascular smooth muscle cell sensitivity to NO. (a) Sodium nitroprusside (SNP) dose-response curve of WT mice treated with telmisartan for 2 weeks in drinking water. Sensitivity to SNP, a NO donor, is not affected in WT aortic vessels treated with telmisartan as depicted by no changes in the (b) EC50 of SNP. (c) L-NAME addition prior to PE constriction enhances basal contractile response of aortas of WT mice treated with telmisartan for 2 weeks. All results are presented as mean ± SEM; with unpaired t-test for two groups where *p<0.05, with n=5 for all groups.

## Slide 5
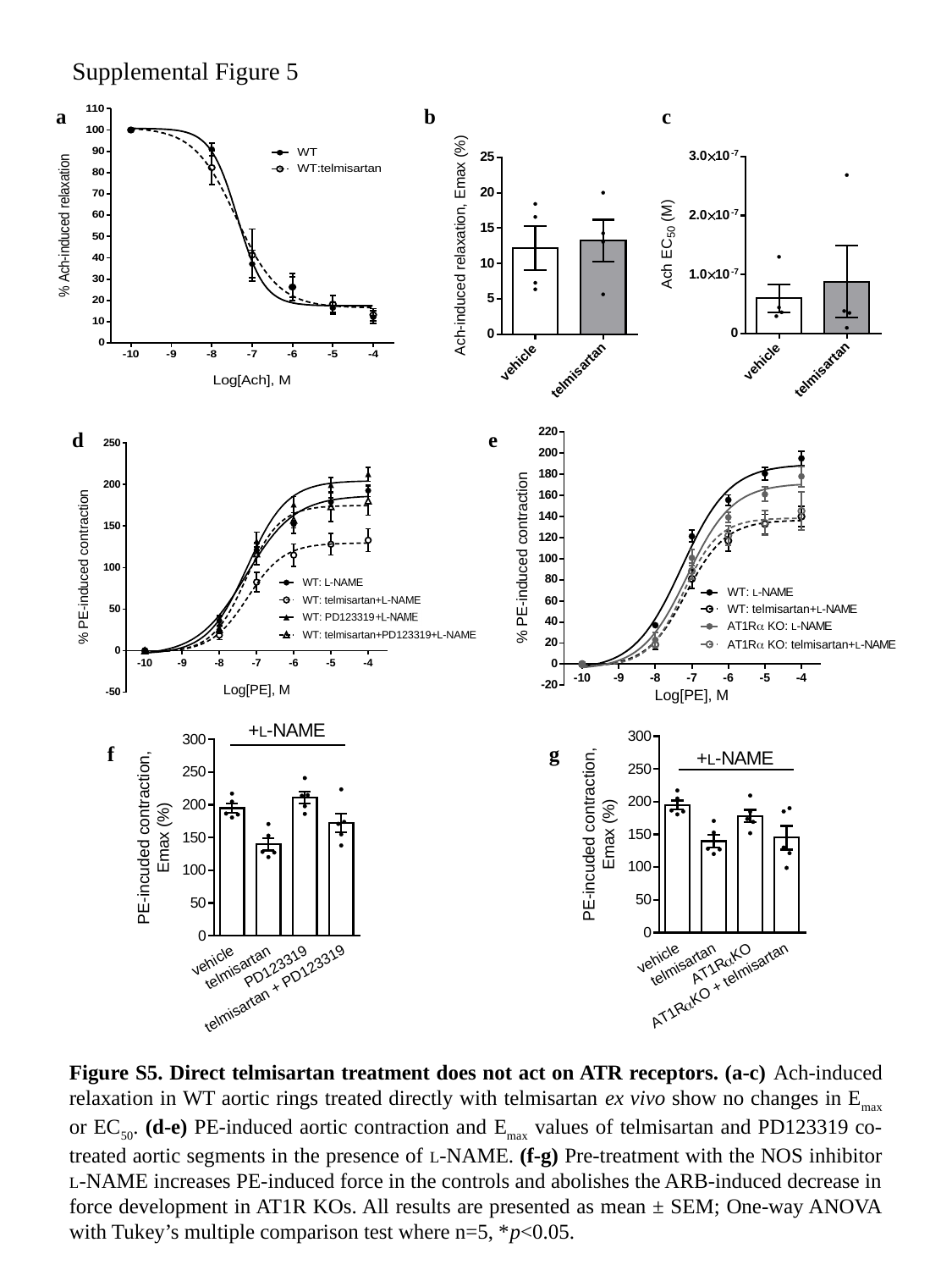

Supplemental Figure 5
a
b
c
d
e
f
g
Figure S5. Direct telmisartan treatment does not act on ATR receptors. (a-c) Ach-induced relaxation in WT aortic rings treated directly with telmisartan ex vivo show no changes in Emax or EC50. (d-e) PE-induced aortic contraction and Emax values of telmisartan and PD123319 co-treated aortic segments in the presence of L-NAME. (f-g) Pre-treatment with the NOS inhibitor L-NAME increases PE-induced force in the controls and abolishes the ARB-induced decrease in force development in AT1R KOs. All results are presented as mean ± SEM; One-way ANOVA with Tukey’s multiple comparison test where n=5, *p<0.05.

## Slide 6
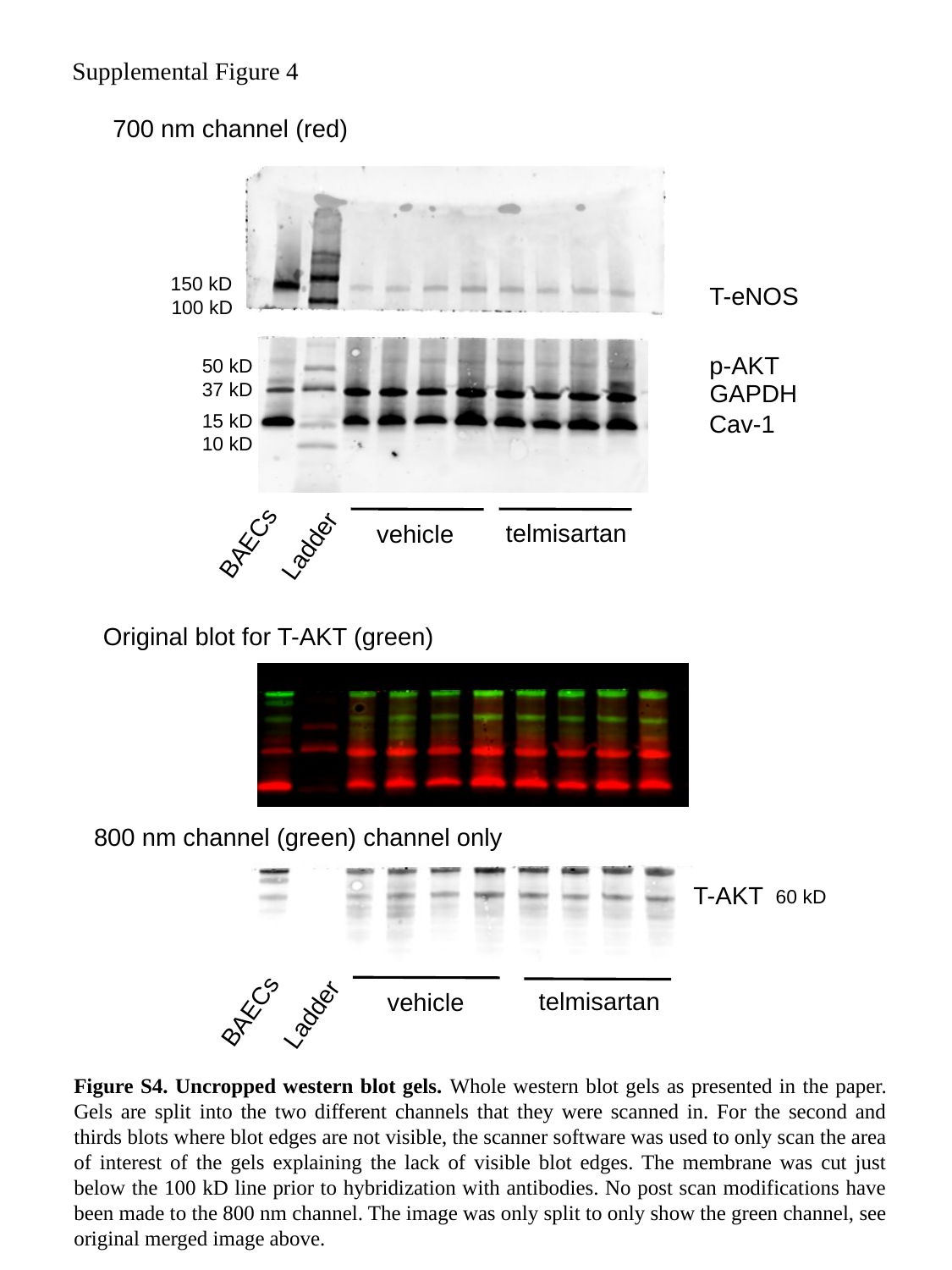

Supplemental Figure 4
700 nm channel (red)
150 kD
T-eNOS
100 kD
p-AKT
50 kD
37 kD
GAPDH
Cav-1
15 kD
10 kD
telmisartan
vehicle
BAECs
Ladder
Original blot for T-AKT (green)
800 nm channel (green) channel only
T-AKT
60 kD
telmisartan
vehicle
BAECs
Ladder
Figure S4. Uncropped western blot gels. Whole western blot gels as presented in the paper. Gels are split into the two different channels that they were scanned in. For the second and thirds blots where blot edges are not visible, the scanner software was used to only scan the area of interest of the gels explaining the lack of visible blot edges. The membrane was cut just below the 100 kD line prior to hybridization with antibodies. No post scan modifications have been made to the 800 nm channel. The image was only split to only show the green channel, see original merged image above.

## Slide 7
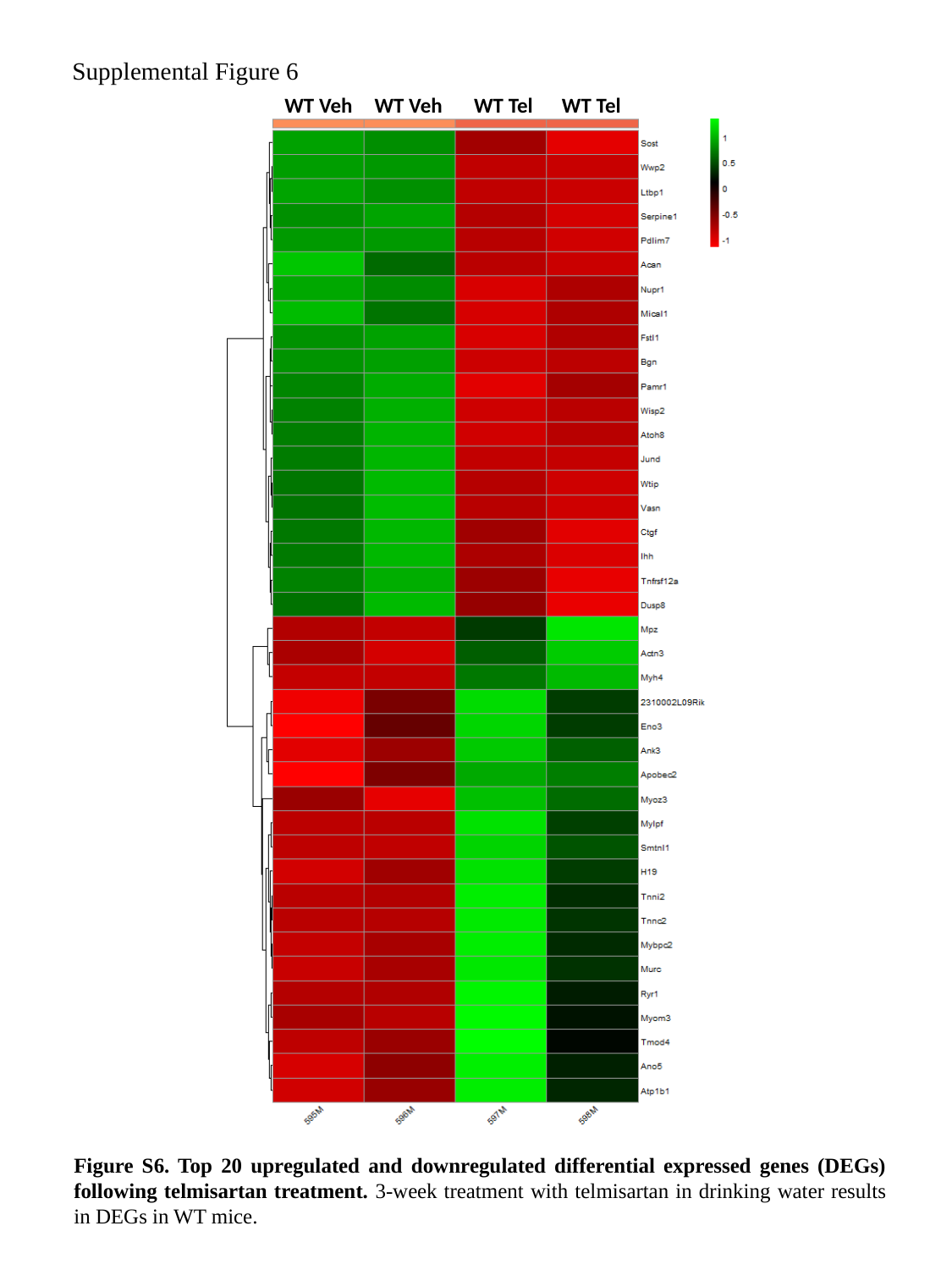

Supplemental Figure 6
WT Veh
WT Veh
WT Tel
WT Tel
Figure S6. Top 20 upregulated and downregulated differential expressed genes (DEGs) following telmisartan treatment. 3-week treatment with telmisartan in drinking water results in DEGs in WT mice.
